# Effect of Angiotensin II on the Left Ventricular Function in a Near-Term Fetal Sheep with Metabolic Acidemia

**DOI:** 10.1155/2011/634240

**Published:** 2011-10-31

**Authors:** Ganesh Acharya, James C. Huhta, Mervi Haapsamo, Ole-Jakob How, Tiina Erkinaro, Juha Räsänen

**Affiliations:** ^1^Women's Health and Perinatology Research Group, Department of Clinical Medicine, Faculty of Health Sciences, University of Tromsø and Department of Obstetrics and Gynecology, University Hospital of Northern Norway, 9038 Tromsø, Norway; ^2^All Children's Hospital, St. Petersburg, FL 33701, USA; ^3^Department of Obstetrics and Gynecology, Oulu University Hospital, 90014 Oulu, Finland; ^4^Cardiovascular Research Group, Department of Clinical Medicine, Faculty of Health Sciences, University of Tromsø, 9037 Tromsø, Norway; ^5^Department of Anesthesiology, Oulu University Hospital, 90220 Oulu, Finland; ^6^Department of Obstetrics and Gynecology, Kuopio University Hospital and University of Eastern Finland, 70211 Kuopio, Finland; ^7^Department of Obstetrics and Gynecology, Oregon Health Sciences University, Portland, OR 97201, USA

## Abstract

We tested the hypothesis that, in acute metabolic acidemia, the fetal left ventricle (LV) has the capacity to increase its contractility in response to angiotensin II infusion. Eleven ewes and their fetuses were instrumented at 127–138/145 days of gestation. The effect of angiotensin II on fetal LV function was assessed using intraventricular pressure catheter and tissue Doppler imaging (TDI). Angiotensin II increased fetal arterial blood pressure, whereas pH and pO_2_ decreased. The heart rate and systemic venous pressure were not affected significantly. The LV end-diastolic and end-systolic pressures, as well as dP/dt_max_, increased. The TDI-derived LV longitudinal myocardial isovolumic contraction velocity and its acceleration and velocity during early filling were higher than those at baseline. The incidence of absent isovolumic relaxation velocity was greater during angiotensin II infusion. In summary, during acute metabolic acidemia, the fetal left ventricle could increase its contractility in response to inotropic stimulus even in the presence of increased afterload. The diastolic LV function parameters were altered by angiotensin II.

## 1. Introduction

Experimental studies have shown that sheep fetuses with increased placental vascular resistance and acute metabolic acidosis are able to maintain right and left ventricular (LV) cardiac outputs [1]. However, they show signs of impaired myocardial contractility during the isovolumic contraction phase and impaired relaxation during the isovolumic and early diastolic filling phases of the cardiac cycle. The global fetal cardiac function is preserved during moderate acidemia despite reduced myocardial contractility [1]. 

Angiotensin II is an important short- and long-term regulator of blood pressure. It is evident that angiotensin II, in addition to its peripheral vasoconstrictive effect, has positive inotropic and chronotropic effects on the heart independent of arterial blood pressure [2]. However, the inotropic response to angiotensin II in cardiac muscle can vary; the responsiveness seems to be greater in the normal healthy myocardium than in the failing muscle [3]. In fact, in adult dogs with pacing-induced heart failure, angiotensin II caused a direct depression in the LV contraction and relaxation and exacerbated the reduced myocyte contractile performance [4]. In addition, myocardial tissue preparations have shown altered responses to angiotensin II after acute myocardial infarction [5]. In humans, several pregnancy complications including placental insufficiency are associated with fetal hypoxia and metabolic acidosis. Furthermore, asphyxiated term infants commonly have biochemical and echocardiographic evidence of abnormal cardiac function [6], and tissue Doppler imaging (TDI) appears to be more sensitive than conventional measurement of fractional shortening in early detection of myocardial dysfunction induced by perinatal asphyxia [6, 7]. In the present acute experimental model on near-term sheep fetuses, we tested the hypothesis that in acute fetal metabolic acidemia LV has the capacity to increase its contractility during angiotensin II infusion. Specifically, we asked the following questions: (1) does angiotensin II infusion increase LV pressure generation and contractility measured by an intraventricular pressure catheter and (2) does angiotensin II infusion affect tissue Doppler-derived parameters of the LV systolic and diastolic function in the presence of increased ventricular afterload?

## 2. Materials and Methods

Eleven ewes of Finnish breed with time-dated pregnancies between 127 and 138 days of gestation (term gestation 145 days) were included in this study. All experiments were performed in accordance with the guidelines of the European Convention for the Protection of Vertebrate Animals Used for Experimental and Other Scientific Purposes (1986) and in compliance with the European Union Directive 86/609/EEC (1997). The research protocol was approved by the Animal Care and Use Committee of the University of Oulu, Finland.

Before surgery, food was withdrawn for 18 hours. Intramuscular ketamine (2 mg/kg) and midazolam (0.2 mg/kg) were given for premedication half an hour before anesthesia. Left external jugular vein was cannulated for intravenous access, and Ringer's lactate solution was infused at a rate of 200 mL/hour. General anesthesia was induced with intravenous propofol (4–7 mg/kg). The anesthesia was maintained with isoflurane (1.5–2.5%) in a 40% oxygen-air mixture delivered via an endotracheal tube. Mechanical ventilation was maintained with a Siemens 730 ventilator (Siemens-Elema AB, Solna, Sweden). Tidal volume was adjusted to 10 mL/kg and respiratory rate to 18/minute. Maternal femoral artery was cannulated to measure the arterial blood pressure (BP), heart rate, and acid-base status. 

A midline laparotomy was performed and a fetal hindlimb was delivered through a small uterine incision. 4F polyurethane catheters (BD Careflow, Becton Dickinson Medical Systems, Singapore) were introduced into the fetal inferior vena cava and descending aorta via femoral vein and artery, respectively. The fetal limb was returned into the uterus, and the uterine incision was closed with a purse-string suture.

A separate small incision was made on the uterus to access the fetal neck. Right carotid artery was dissected, and a 5F cannula with a back flow valve was introduced using a guide wire and introducer. A 3F Millar SPR877 catheter (Millar Instruments, Houston, Tex, USA) was advanced into the fetal LV through the 5F cannula. The position of the catheter within the LV cavity was confirmed by ultrasonography. The micromanometer pressure transducer output was fed to a custom-built amplifier and connected to a signal-processing unit (Sigma 5DF, Cardiodynamics corp.) for the continuous measurement of LV pressure.

Maternal arterial BP, heart rate, and oxygen saturation (SaO2) and fetal BP, heart rate, and central venous pressure (CVP) were recorded continuously at a 100 Hz sampling rate using a polygraph (UIM100A, Biopac Systems Inc., Santa Barbara, Calif, USA) and computerized data acquisition software (Acqknowledge v. 3.5.7 for Windows, Biopac Systems Inc., Santa Barbara, Calif, USA). Acid-base status was checked (corrected to 39°C) at the end of baseline and angiotensin II phases using an Abbot i-sat 1 arterial blood gas analyser (i-Stat, East Windsor, NJ, USA). 

Ultrasonography was performed using the Vivid 7 Dimension ultrasound system (GE Vingmed Ultrasound, Horten, Norway) with a 10 MHz-phased array transducer through the uterine wall. Mitral valve**'**s blood flow velocity waveforms were obtained using pulsed-wave Doppler to measure the maximum velocity of the blood flow during early filling (E) of the LV. Longitudinal myocardial velocities were recorded from the LV lateral wall at the level of mitral valve annulus using pulsed-wave tissue Doppler imaging (TDI) with the sample volume (1–1.5 mm) aligned parallel to the myocardial wall (insonation angle <15 degrees) at a sweep speed of 100 mm/s. All the ultrasonographic examinations were performed by a single investigator.

**Figure 1:**
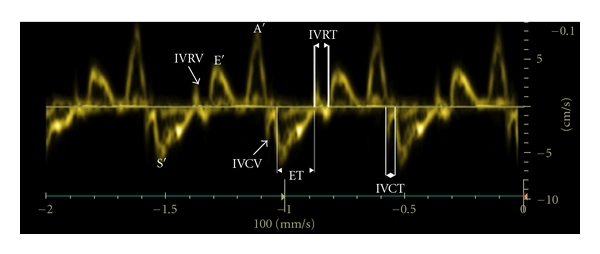
Tissue Doppler-derived left ventricular longitudinal myocardial velocities at the level of mitral valve annulus obtained from a near-term sheep fetus. Isovolumic contraction velocity (IVCV), isovolumic contraction time (IVCT), velocity during ventricular systole (S′), isovolumic relaxation velocity (IVRV), isovolumic relaxation time (IVRT), ejection time (ET), velocities during early ventricular filling (E′), and atrial contraction (A′) phases of the cardiac cycle.

Tissue Doppler recordings were analyzed offline using dedicated software (EchoPac PC v.6.1.2, GE Medical Systems). The LV maximal longitudinal myocardial velocities were measured during the isovolumic contraction (IVCV), ventricular systole (S′), isovolumic relaxation (IVRV), early ventricular filling (E′), and atrial contraction (A′) phases of the cardiac cycle (Figure 1). The isovolumic myocardial acceleration and deceleration were calculated by dividing the peak IVCV and IVRV by the time intervals from the onset to the peak of these velocity waveforms, respectively [1]. The LV isovolumic contraction (IVCT) and relaxation times (IVRT) were measured, and their proportions (%) of the total cardiac cycle were calculated as described previously [1]. To improve measurement precision, the sweep speed of the Doppler recordings was adjusted according to the fetal heart rate (i.e., increased to 200 mm/s if the fetal heart rate exceeded 160 beats/min) during offline analysis.

Following baseline measurements, 4 mL of angiotensin II solution (1.67 mcg/mL) was diluted with 96 mL of 0.9% saline and infused into the fetal inferior vena cava at a rate of 100–200 ml/hour (adjusted to keep the fetal MAP increased at 15–20 mmHg above the baseline) and all the above measurements were repeated. The angiotensin II infusion was continued for approximately 20 minutes until all the measurements were made. At the end of the experiment, the animals were euthanized with an intravenous overdose (1 mg/kg) of pentobarbital sodium. Fetal weight was postmortem determined. Data were analysed using Statistical Software for Social Sciences for windows version 16.0 (SPSS Inc. Chicago, Ill, USA). To examine differences between the baseline and the angiotensin II phase, the paired sample *t*-test was used for continuous parametric variables and the Fisher's exact test for categorical variables. Statistical significance level was set at a *P* value ≤ 0.05.

## 3. Results

**Figure 2:**
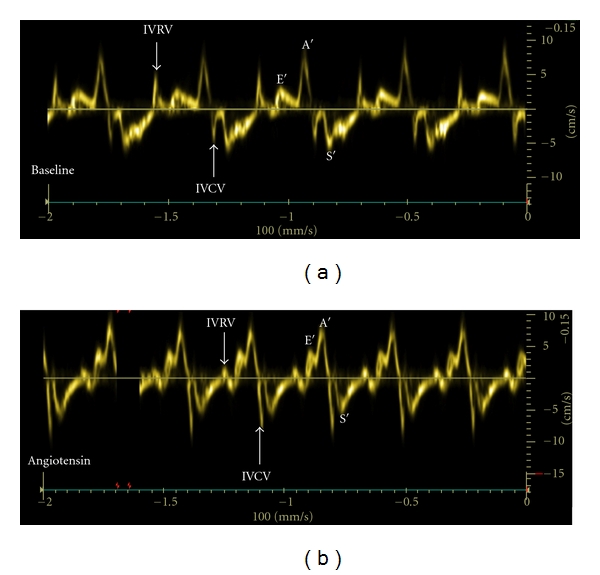
Tissue Doppler-derived left ventricular longitudinal myocardial velocities at the level of mitral valve annulus at baseline (a) and during angiotensin II infusion (b). Note an increase in myocardial isovolumic contraction (IVCV) and early ventricular filling (E′) velocities and a decrease in myocardial isovolumic relaxation velocity (IVRV) during angiotensin II infusion.

**Figure 3:**
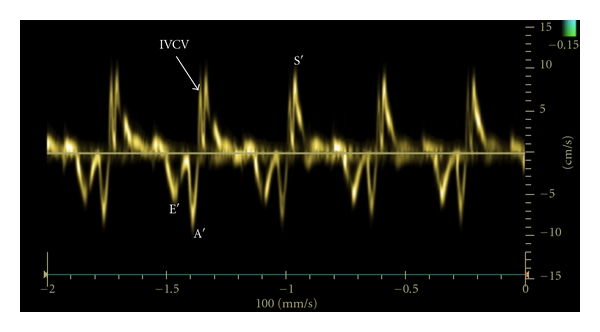
Tissue Doppler-derived left ventricular longitudinal myocardial velocities at the level of mitral valve annulus during angiotensin II infusion demonstrating absence of myocardial isovolumic relaxation velocity (IVRV).

The mean body weight of the ewes was 67 (range, 52–84) kg and the mean fetal weight was 2787 (range, 2090–3700) g. The mean gestational age was 132 days. Maternal blood pressures and acid-base values remained unchanged during the experiment (Table 1). During angiotensin II infusion fetal systolic, mean, and diastolic blood pressures increased significantly. Fetal heart rate and systemic venous pressure were not affected by angiotensin II infusion. Fetal pH and pO_2_ values decreased significantly during the experiment (Table 2). There was almost a two-fold increase in LV dP/dt_max_ (*P* < 0.003) during angiotensin II infusion. In addition, LV end-diastolic and end-systolic pressures increased significantly (Table 3). Angiotensin II infusion significantly increased TDI-derived LV IVCV and E′ velocity (Figure 2). In addition, the LV isovolumic myocardial acceleration demonstrated over a two-fold increase (*P* < 0.02) during angiotensin II infusion. The LV E/E′ ratio decreased (*P* < 0.02). The incidence of absent IVRV was higher (*P* < 0.02) during angiotensin II infusion (Figure 3). LV IVCT% and IVRT% did not change significantly during the experiment (Table 4).

## 4. Discussion

The rationale for performing this experimental study was to investigate the functional capacity and reserve of the fetal LV during acute metabolic acidemia. We demonstrated that in the fetal sheep at near-term gestation the LV was able to increase its contractility in response to angiotensin II infusion despite fetal acidemia and increased cardiac afterload. A positive response to inotropic stimulus may indicate that the myocardial dysfunction is transient, and there is a potential for recovery whereas the chances of recovery may be poor when the response is negative. Angiotensin II was chosen because it has a positive inotropic effect on the heart, and it is a potent peripheral vasoconstrictor. In the fetal sheep, it increases myocardial blood flow and cardiac output despite a significant increase in afterload [8].

We found that during angiotensin II infusion the fetal LV dP/dt_max_, end-diastolic and end-systolic pressures as well as the arterial blood pressures increased significantly, but the fetal central venous pressure increase was not significant. These findings are in agreement with published experimental studies on several different adult animal species. Angiotensin II is known to increase the venous return, and, by this mechanism, it can lead to elevated preload and left ventricular end-diastolic pressure [9, 10]. In addition, increased LV dP/dt_max_ suggests that angiotensin II infusion improved ventricular contractility. Even though dP/dt_max_ is relatively insensitive to alterations in afterload, it can be affected by changes in preload [11, 12]. This could partially explain the increase in LV dP/dt_max_.

One of the main findings of the present study is that during angiotensin II infusion TDI-derived LV longitudinal IVCV and its acceleration increased significantly. Both of these indices describe preejection events in the myocardium and, thus, are less influenced by afterload than ejection phase indices. In fact, experimental animal studies have shown that the isovolumic myocardial acceleration is independent of cardiac loading conditions [13]. Our results demonstrate that fetal LV can increase its contractility in the presence of fetal metabolic acidemia and elevated cardiac afterload. This is also supported by the unchanged IVCT% during the angiotensin II infusion. The IVCT characterizes the period that is needed for the ventricle to increase its pressure from an atrial to a systemic level. During angiotensin II infusion, the pressure gradient between LV end-diastolic pressure and arterial diastolic blood pressure was greater than at baseline suggesting that the LV was able to improve its pressure generation. Experimental studies on adult pigs under normoxemic conditions have shown that angiotensin II infusion has a positive inotropic effect on the LV independent of arterial blood pressure levels [2]. However, the inotropic response seems to vary, being greater in the healthy myocardium than in the failing muscle [3]. In fact, it has been demonstrated that tachycardia-induced heart failure alters LV and myocyte responses to angiotensin II, so that angiotensin II produces direct depression of LV contractility and exacerbates myocyte contractile dysfunction [4]. Altogether, our study suggests that, in metabolic acidemia, fetal LV can increase its contractility in response to an inotropic stimulus even in the presence of increased afterload demonstrating the systolic functional reserve of the fetal LV. The second important finding of our experimental study is that during angiotensin II infusion IVRV was absent significantly more often than at baseline condition. Previously, we have shown that acute fetal metabolic acidemia decreases myocardial lengthening velocity during isovolumetric relaxation [1] suggesting that metabolic acidosis adversely affects the calcium- and energy-dependent active myocardial relaxation. Significant metabolic acidosis can lead to depletion of myocardial glycogen and ATP stores that are associated with impaired repolarization. In addition, angiotensin II itself seems to have a detrimental effect on isovolumetric cardiac diastolic function possibly by a decrease in Ca^2+^ efflux through the Na^+^/Ca^2+^ exchanger produced by the angiotensin-II-induced prolongation of the action potential duration [14]. We suggest that the early sign of fetal cardiac diastolic dysfunction could be diminished myocardial lengthening velocity during isovolumetric relaxation period. When diastolic function further deteriorates, isovolumetric myocardial lengthening velocity disappears. In the present study, IVRT% did not change during the angiotensin II infusion. IVRT represents the time interval that is needed for the ventricle to decrease its pressure from a systemic to an atrial level. It seems that IVRT% is not as sensitive indicator of myocardial diastolic function as myocardial lengthening velocity, and IVRT% can be more affected by ventricular loading conditions than the myocardial movement itself. Disturbances in diastolic function often precede the changes in global cardiac systolic performance, and the assessment of cardiac diastolic function could be useful in monitoring fetuses at risk for cardiac dysfunction. Our present study suggests that the absence of IVRV could be an early sign of abnormal ventricular diastolic function in the fetus.

 In the present study, left ventricular E′-wave velocity increased significantly during angiotensin II infusion. E′-wave velocity is used as an index of active ventricular relaxation, but it is also sensitive to changes in ventricular loading conditions. Our results demonstrate that angiotensin II infusion increased fetal LV preload, and we suggest that increased E′-wave velocity mainly reflected elevated ventricular preload. However, as acidemia with increased afterload is known to decrease mitral E′-wave velocity [1], it could be argued that angiotensin II overcomes the negative effect of acidosis on myocardial relaxation during early ventricular filling. This is also supported by the fact that E/E′ ratio was lower during angiotensin II infusion compared to baseline despite increased LV end-diastolic pressure. However, in the fetus, the ventricular filling occurs mainly during the atrial contraction rather than in early diastole, and E/E′ ratio may not reflect LV end-diastolic pressure as in adults [15]. LV myocardial velocity during atrial contraction (A′-wave velocity) did not change significantly during angiotensin II infusion. This could suggest that angiotensin II did not significantly augment atrial contraction. However, A′-wave velocity is also affected by changes in ventricular loading conditions. LV peak S′-wave velocity describes myocardial shortening during the ejection phase of the systole. In adults, S′-wave velocity correlates with ventricular ejection fraction, and it has been used as an index of cardiac systolic function [16]. In the present study, we found no significant increase in this parameter, despite significantly improved LV contractility. Myocardial S′-wave velocity is sensitive to changes in the afterload. Increased systemic arterial blood pressure and LV afterload could have blunted the positive inotropic effect of angiotensin II on myocardial S′-wave velocity. It appears that these load-dependent parameters of myocardial lengthening and shortening may not be as useful in the evaluation of fetal cardiac function as in adults.

The present study has certain limitations. The experiments were performed under general anesthesia. Isoflurane may modify fetal cardiovascular regulation. However, studies on newborn lambs under isoflurane anesthesia have shown that lambs can increase cardiovascular performance during stress [17]. We used an acute animal preparation in order to acquire intraventricular pressure measurements simultaneously with TDI. The fetuses were acidemic at baseline as a result of surgical intervention, manipulation, and instrumentation. As acute acidemia may alter fetal hemodynamic, metabolic, and endocrine responses [18], it can be argued that some of the changes in the left ventricular function observed in our sheep fetuses following angiotensin II infusion may have been caused by possible release of catecholamines. Although fetal plasma catecholamine levels were not measured, the mean values of the load-independent TDI parameters measured at baseline in the present study were similar to those obtained in our previous study with chronic animal preparation during comparable fetal metabolic acidemia demonstrating the validity of our experimental model [1]. Care was taken to minimize methodological errors related to TDI measurements. However, a fetal electrocardiogram was not obtained simultaneously with tissue Doppler recording. Although different phases of cardiac cycle can be easily identified on a myocardial tissue Doppler's velocity envelope, a simultaneous electrocardiogram could improve precision. The sample volume was placed accurately at the level of mitral valve annulus, and the angle of insonation was kept <15 degrees in all cases during repeated measurements. The highest available frame rates were used when obtaining TDI-derived measurements. Finally, all the TDI measurements were obtained by a single investigator. In human fetuses, intraobserver variability of TDI-derived myocardial velocity measurements has been shown to be comparable to pulse Doppler-derived parameters [19].

In conclusion, by using an acute experimental fetal sheep model at near-term gestation, we demonstrated that, in metabolic acidemia, the fetal LV can increase its contractility in response to inotropic stimulus even in the presence of increased afterload demonstrating the systolic functional reserve of the fetal LV. However, LV diastolic function during the isovolumic phase was disturbed by angiotensin II infusion.

## Figures and Tables

**Table 1:** Invasively monitored maternal hemodynamic parameters and acid-base status at baseline and during fetal angiotensin II infusion. Data are presented as mean (SD).

	Baseline	Angiotensin II	*P* value
Heart rate, beats/min	98 (15)	98 (19)	0.807
Systolic BP, mmHg	95 (7)	94 (109)	0.283
Diastolic BP, mmHg	64 (9)	62 (11)	0.460
Mean arterial pressure, mmHg	74 (8)	72 (11)	0.402
Oxygen saturation, %	95 (4.5)	94 (4.4)	0.115
pH	7.36 (0.03)	7.37 (0.02)	0.115
Base excess, mmol/L	−3.18 (3.2)	−2.36 (2.1)	0.203
PCO_2_, kPa	5.13 (0.92)	5.13 (0.43)	0.990
PO_2_, kPa	14.5 (4.3)	13 (4.1)	0.033
Lactate, mmol/L	0.64 (0.22)	0.65 (0.24)	0.832

**Table 2:** Invasively monitored fetal hemodynamic parameters and acid-base status at baseline and during angiotensin II infusion. Data are presented as mean (SD).

Parameter	Baseline	Angiotensin II	*P* value
Heart rate, beats/min	153 (41)	170 (40)	0.337
Systolic BP, mmHg	55 (8)	83 (18)	<0.001
Diastolic BP, mmHg	38 (5)	56 (12)	<0.001
Mean arterial pressure, mmHg	43 (6)	65 (14)	<0.001
Central venous pressure, mmHg	9 (2)	14 (13)	0.327
pH	7.11 (0.12)	7.04 (0.16)	0.003
Base excess, mmol/L	−9.4 (5.0)	−11.7 (6.96)	0.029
PCO_2_, kPa	8.6 (2.3)	9.3 (2.3)	0.020
PO_2_, kPa	2.4 (0.9)	1.5 (0.8)	0.004
Lactate, mmol/L	7.6 (3.7)	7.4 (2.8)	0.701

**Table 3:** Fetal left ventricular pressures at baseline and during angiotensin II infusion. Data are presented as mean (SD).

	Baseline	Angiotensin II	*P* value
dP/dt_max_, mmHg/s	1224 (330)	2030 (476)	0.003
End-systolic pressure, mmHg	64 (18)	93 (26)	0.001
End-diastolic pressure, mmHg	14 (6)	20 (9)	0.005

**Table 4:** Fetal left ventricular tissue Doppler-derived parameters at baseline and during angiotensin II infusion. Data are presented as mean (SD) or *n* (%).

	Baseline	Angiotensin II	*P* value
IVCV, cm/s	4.1 (2.3)	6.4 (2.4)	0.039
IVCVAccel, cm/s^2^	350 (250)	780 (490)	0.022
S′-velocity, cm/s	4.7 (1.5)	5.7 (2.4)	0.165
IVRV, cm/s	2.2 (1.4)	0.6 (1.5)	0.15
Absent IVRV, *n* (%)	2 (20)	8 (80)	0.024
E′-velocity, cm/s	3.1 (0.74)	4.4 (0.83)	0.015
A′-velocity, cm/s	8.2 (4.0)	12.9 (6.5)	0.073
E/E′ ratio	8.7 (2.0)	5.7 (2.1)	0.015
IVCT%	10.4 (4.2)	12.6 (4.8)	0.166
IVRT%	13.3 (7.8)	12.3 (6.1)	0.745

IVCV, isovolumic contraction velocity; IVCVAccel, isovolumic myocardial acceleration; S′-velocity, myocardial velocity during the left ventricular systole; IVRV, isovolumic relaxation velocity; E′-velocity, myocardial velocity during early ventricular filling; A′-velocity, myocardial velocity during atrial contraction; IVCT, isovolumic contraction time; IVRT, isovolumic relaxation time.
